# New Insights and Experimental Investigation of High-Temperature Gel Reinforced by Nano-SiO_2_

**DOI:** 10.3390/gels8060362

**Published:** 2022-06-08

**Authors:** Hongbin Guo, Jijiang Ge, Longjie Li, Guoliang Zhang, Ziwei Li, Wenhui Wang, Mingjia Liu

**Affiliations:** 1 School of Petroleum Engineering, China University of Petroleum (East China), Qingdao 266580, China; 15588674096@163.com (H.G.); lilongjie0809@163.com (L.L.); wangwh1221@163.com (W.W.); liumingjia037@163.com (M.L.); 2 Research Institute of Exploration and Development, Tarim Oilfield Company, PetroChina, Korla 841000, China; dz17667749529@126.com; 3 Petroleum Engineering and Technology Institute, SINOPEC Jiangsu Oilfield Company, Yangzhou 225009, China; liziwei0728@163.com

**Keywords:** reinforced high-temperature gel, HPAM, water-soluble phenolic resin, nanoparticles, syneresis

## Abstract

The properties of a reinforced gel with partially hydrolyzed polyacrylamide (HPAM) as the main agent, water-soluble phenolic resin (WSPR) as the crosslinker, and nano-SiO_2_ as the stabilizer were evaluated in terms of gelation time, gel strength and thermal stability under the conditions of 110 °C and 12.124 g/L salinity in water. The results showed that the gelation time of the gel with high strength was adjustable from 3 to 23 h, remaining stable for more than 180 days under stratigraphic conditions, although with a certain degree of early dehydration in the gel. Cryo-scanning electron microscopy (cryo-SEM) and dynamic light scattering (DLS) analysis revealed that nano-SiO_2_ improves the dispersion of the polymer in water, resulting in a more homogeneous structure of the formed gel and thus improving the strength of the gels. In addition, rheological tests and cryo-SEM showed that the interaction between nano-SiO_2_ and the polymer could inhibit the degradation of polymer to a certain extent and improve the thermal stability of the gel. However, the oxidative degradation of the gel is still the main cause of early dehydration of water-soluble phenolic resin gel, and the addition of a small amount of hydroquinone to the gelants can significantly improve the antioxidative degradation properties of phenolic resin gel.

## 1. Introduction

Polymer gels have become one of the most commonly used materials in the oil industry for conformance control and enhanced recovery. Over the years, they have been successfully applied to reservoirs at a variety of temperatures (40–150 °C) under a variety of salinity conditions with alternative performance and broad environmental adaptability [1,2,3,4,5]. The polymer gels that can be applied to high-temperature reservoirs are mainly organic crosslinked gels, including phenolic gels, polyethyleneimine gels, etc. [3,4,6] Phenolic gels were developed first and adapted to a wide range of temperatures and salinities. In the early 1980s, Falk, Swanson, et al., proposed the preparation of gels using phenol and formaldehyde cross-linked with polyacrylamide [7,8]. However, phenol and formaldehyde are toxic, so Ahmad Moradi-Araghi proposed the use of hexamethylenetetramine (HMTA) instead of formaldehyde and salicylamide, salicylic alcohol, salicylic acid, furfuryl alcohol, and hydroquinone (HQ) instead of phenol for the preparation of temperature- and salinity-resistant gels [9,10,11,12,13]. Subsequently, HQ and HMTA gradually became the most commonly used compound crosslinkers for the preparation of high-temperature gels [3,14]. Eriksen et al. [15] prepared stable gels aged in seawater at 120 °C for 30 days with almost no syneresis using copolymers synthesized from acrylamide (AM) and acrylamide-2-methylpropane sulfonic acid (AMPS) monomers crosslinked with phenol and HMTA. High-temperature stable gels were also prepared by Dovan, Hutchins, et al., using cationic polymers crosslinked with HMTA and HQ in a vacuum-deoxygenated and nitrogen-filled environment [6,16]. The prepared gel can be stable for 12 months at 149 °C and 5 months at 176.7 °C in seawater and has been successfully applied in the field several times in New Mexico, the Gulf of Mexico, the North Sea, and the Maracaibo Basin of Venezuela [5,6,16]. In addition, water-soluble phenolic resin (WSPR) is also a good alternative to phenolic crosslinkers with low toxicity, with the benefits of lower cost and easy transportation and application. Water-soluble phenolic resin is generated by the addition reaction of formaldehyde and phenol under alkaline conditions to produce further condensation products, hydroxymethyl functional group of which is strongly reactive and can be condensed with the amide group on the polymer molecular chain to form a gel [17].

In recent years, the incorporation of nanomaterials in the development of gel systems has gradually become a conventional method used to enhance gel properties. Numerous studies have shown that the introduction of nanoparticles into polymer gel systems can significantly improve the strength, temperature, and salinity resistance of gels. Wu et al. [18] added 0.1 wt% silica sol with a median particle size of 7 nm to a gelling solution consisting of 0.5 wt% HPAM and 0.5 wt% water-soluble phenolic resin, and the resulting gel was dehydrated at 70 °C for 180 days at a rate of less than 4% (the gel without nanoparticles was significantly dehydrated after 30 days at 70 °C). Dai et al. [19] reported that a gel reinforced by nano-SiO_2_ could withstand temperatures up to 110 °C and salinity up to 212.6 g/L. The mechanism of nanoparticle-reinforced gels has been extensively studied. Analysis of nanoparticle composite gels by differential scanning calorimetry (DSC), dynamic light scattering (DLS), and environmental scanning electron microscopy (ESEM) showed [20,21,22,23] that nanoparticles can adsorb water molecules and hydrated ions through hydrogen bonding and dipole interaction, thus increasing the bound water content in the gels. Moreover, nanoparticles can also form hydrogen bonds with amide groups and other groups, which act as physical crosslinkers [24,25,26,27]. This physical crosslinking substantially retards the hydrolysis of the amide groups and also increases the network density of the gel, which is the main reason for the improved strength and stability of the gel afforded by nanoparticles, as mentioned in recent literature references shown in Table 1.

The phase behavior of nanoparticles and polymers in aqueous solutions is often neglected. Excessive molecular weight of polymer molecules can lead to entanglement and aggregation when dissolved in water, often affecting the gel structure [28] and resulting in a non-uniform gel network. Some studies have also shown that the addition of appropriate nanoparticles to aqueous polymer solutions can change the entanglement state of the polymer in water [17,29,30,31]. Ginzbury et al. [29] found that when the particle size of nanoparticles is smaller than the rotational radius of polymer chains, the nanoparticles can inhibit the interaction between polymer chains and reduce the mixing entropy of the system, resulting in a more homogeneous and stable phase. Therefore, we believe that the addition of nano-SiO_2_ to the gelling solution may affect the dispersion behavior of HPAM in water, which, in turn, affects the network structure and properties of the gel.

Usually, HPAM undergoes rapid hydrolysis at ambient temperatures above 90 °C [32]. This situation is even worse in brine because the carboxylic acid produced by hydrolysis reacts with divalent cations, such as Ca^2+^ and Mg^2+^, causing gel syneresis. [33] Therefore, HPAM is not suitable for high-temperature and high-salinity reservoirs, although the addition of nanoparticles can effectively improve the temperature- and salinity-resistance limit of HPAM. In the present study, enhanced gels were prepared with HPAM, WSPR, and nano-SiO_2_, and their gelling properties were evaluated under the conditions of 110 °C and 12.124 g/L salinity. We further verified nanoparticle–polymer interactions using DLS, rheological testing, and cryo-scanning electron microscopy (cryo-SEM). The results showed that the incorporation of nanoparticles improved the uniformity of polymer dispersion in water and reduced the entanglement and aggregation of polymers. Additionally, the interaction of polymers with nanoparticles can inhibit the degradation of polymers and increase the strength of polymer networks. Furthermore, we found that nanoparticles did not completely inhibit the degradation of polymers, offering a new insight to improve the thermal stability of gels by adding free-radical terminators. We hope that the results of this study will contribute to the development of nanocomposite gels with excellent properties.

## 2. Results and Discussion

### 2.1. Nano-SiO_2_-Reinforced Gel Properties

**Figure 1:**
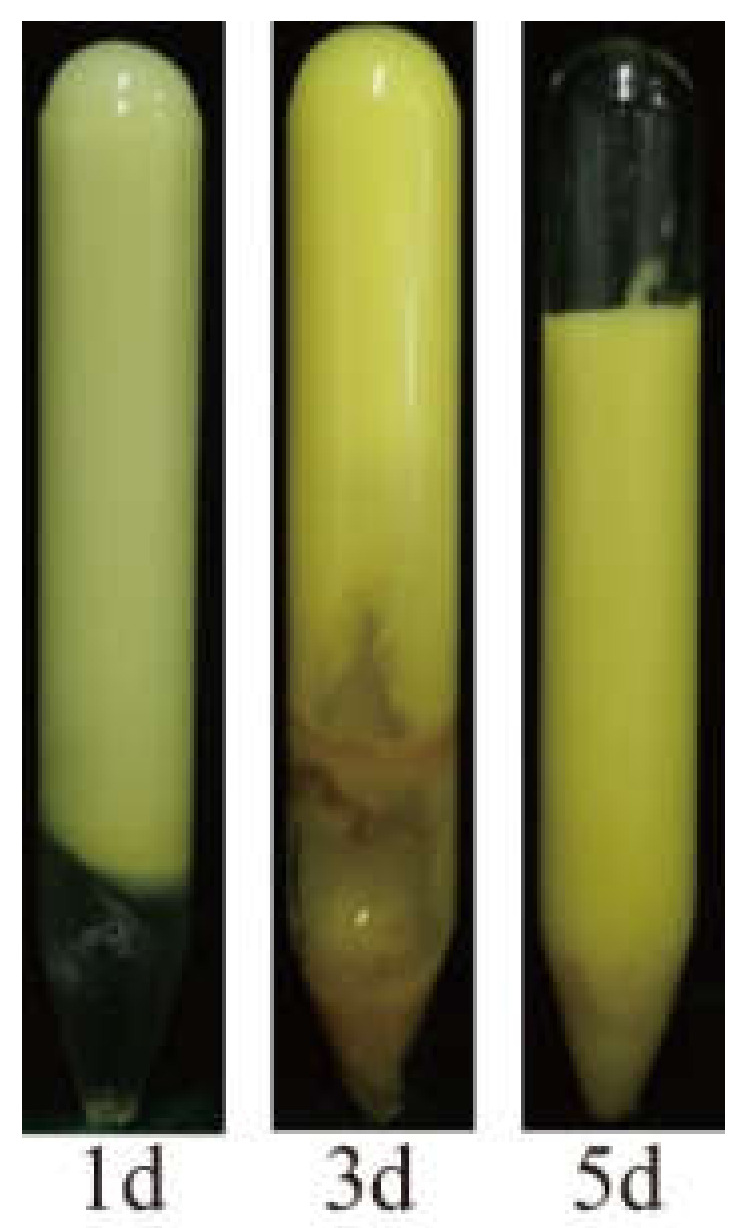
State of the gel prepared from 0.6 wt% HPAM and 0.6 wt% WAPR after aging at 110 °C for 1, 3, and 5 days.

**Figure 2:**
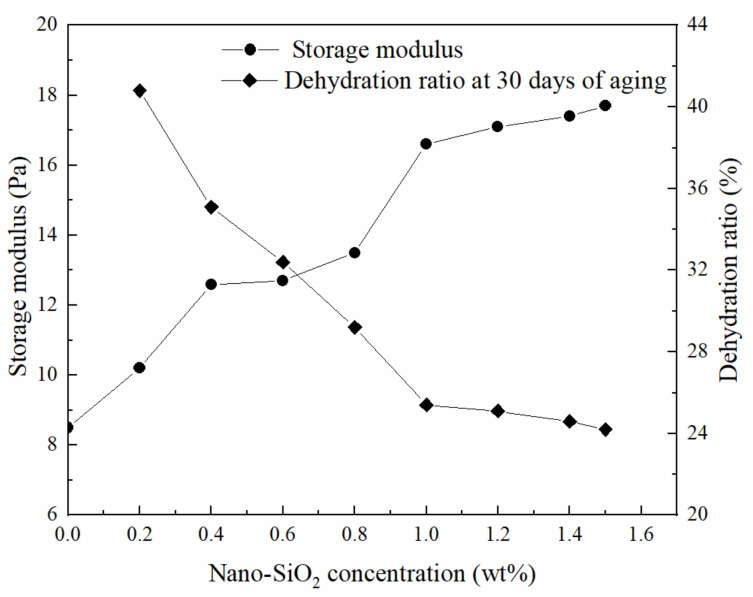
Effect of nano-SiO_2_ dosage on the gel strength and thermal stability of gels containing 0.6 wt% HPAM, 0.6 wt% WSPR, and different concentrations of nano-SiO_2_.

Gelation time, gel strength, and thermal stability are three major indicators for the use of gels as a plugging agent and are related to the injection location, blocking effect, and expiration date of the plugging agent, respectively. We found that the gel containing 0.6 wt% HPAM and 0.6 wt% WSPR prepared with brine of 12.124 g/L salinity broke after aging at 110 °C for 5 days (Figure 1). Incredibly, the stability of the gels obtained by adding a certain amount of nano-SiO_2_ to the gelant was significantly improved. Although there was a certain percentage of dehydration in the gels, the general stability was maintained, as shown in Figure 2.

**Figure 3:**
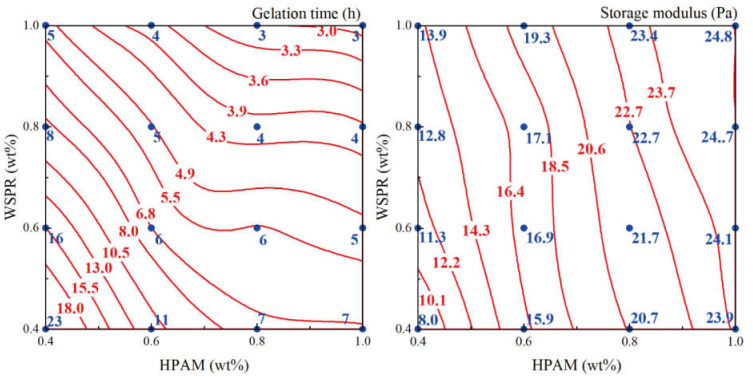
Gelation time and gel strength (storage modulus) of phenolic resin gel at 110 °C.

As the concentration of nano-SiO_2_ was increased, the gel strength increased, and the dehydration ratio decreased. When the concentration of nano-SiO_2_ exceeds 1 wt%, the gel performance gradually reaches the optimum, and the strengthening effect of nano-SiO_2_ on the gel approaches the limit. Compared with the gel without nano-SiO_2_, the storage modulus of the gel with 1 wt% nano-SiO_2_ was doubled, and the dehydration ratio decreased to only 25% after aging at 11 °C for 30 days. Therefore, in the subsequent study, we chose to add 1.0 wt% nano-SiO_2_ to the gelants and further investigated the effect of polymer and crosslinker concentration on the gel properties. The results are shown in Figure 3 (red line and red numbers are fitted values; blue dots and blue numbers are measured values).

**Figure 4:**
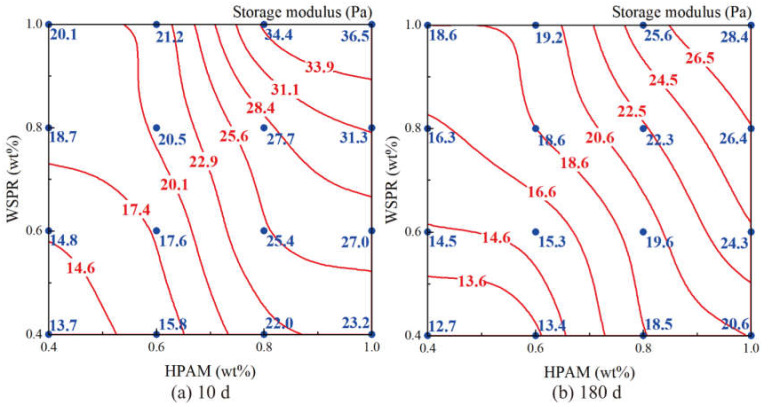
Strength isogram of different component gels aged at 110 °C for different times: (**a**) Strength of gels aged at 110 °C for 10 days; (**b**) Strength of gels aged at 110 °C for 180 days.

**Figure 5:**
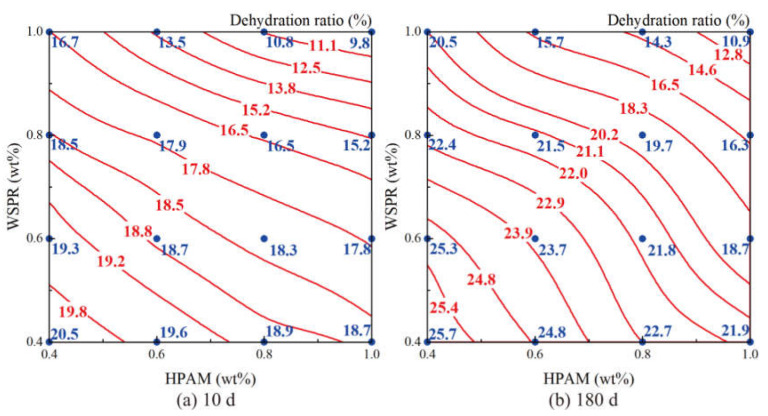
Dehydration ratio isogram of different component gels after aging at 110 °C for different times: (**a**) Dehydration ratio of gels aged at 110 °C for 10 days; (**b**) Dehydration ratio of gels aged at 110 °C for 180 days.

The results showed that the gel prepared by crosslinking HPAM with WSPR had a gelation time of between 3 and 23 h and a gel storage modulus of 8–25 Pa. When the concentration of both HPAM and WSPR in the gel was greater than 0.5 wt%, their storage modulus was greater than 10 Pa, and representing high-strength gels. The storage modulus and dehydration ratio of the above gel at 10 and 180 days of aging at 110 °C are shown in Figure 4 and Figure 5, respectively (red line and red numbers are fitted values; blue dots and blue numbers are measured values).

The gels of different components were able to reach a storage modulus of more than 10 Pa after aging at 110 °C for 10 days, whereas a phenomenon of intense dehydration at the beginning of gel aging was observed, with dehydration ratios ranging from 9.8% to 20.5%. After 180 days of gel aging, the energy storage modulus of the gel only showed a slight decrease, and the dehydration ratio only increased marginally. Therefore, we suggest that high-strength gel can be prepared by using HPAM as the gelling agent, WSPR as the crosslinker, and nano-SiO_2_ as the stabilizer in brine with a salinity of 12.124 g/L at 110 °C.

### 2.2. Mechanism of Nano-SiO_2_-Reinforced Gel

#### 2.2.1. Effect of Nano-SiO_2_ on the Dispersion of Polymers in the Gelant

**Figure 6:**
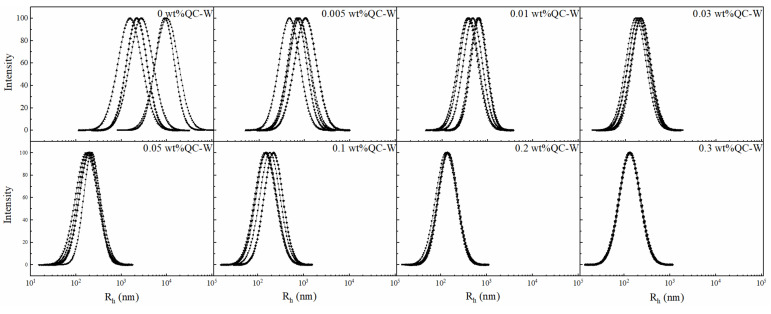
Influence of nano-SiO_2_ on the hydrodynamic radius (R_h_) of HPAM.

The effect of nano-SiO_2_ on the dispersion state of HPAM in water was investigated by DLS to determine the R_h_ of polymers in aqueous solutions containing different concentrations of nano-SiO_2_. The concentration of HPAM was fixed at 0.05 wt% in this study because high concentrations of HPAM would entangle with one another to produce polymer agglomerates with excessive hydrodynamic radii, which would exceed the measurement range of the instrument. The R_h_ distribution of each sample was measured five times and fitted with a laser particle size analyzer to calculate its log-normal distribution; the results are shown in Figure 6.

With an increase in nano-SiO_2_ concentration in the solution, the uneven distribution of the average R_h_ of the polymer, from several hundred to several thousand nm, gradually concentrated to approximately 130 nm, and the large polymer agglomerates were significantly reduced. This indicates that after the introduction of nano-SiO_2_ into HPAM solution, their interaction inhibits the entanglement of the polymer in water and enables the dispersion of polymer molecules in water to achieve a more uniform and stable state.

#### 2.2.2. Effect of Nano-SiO_2_ on Polymer Network Structure

**Figure 7:**
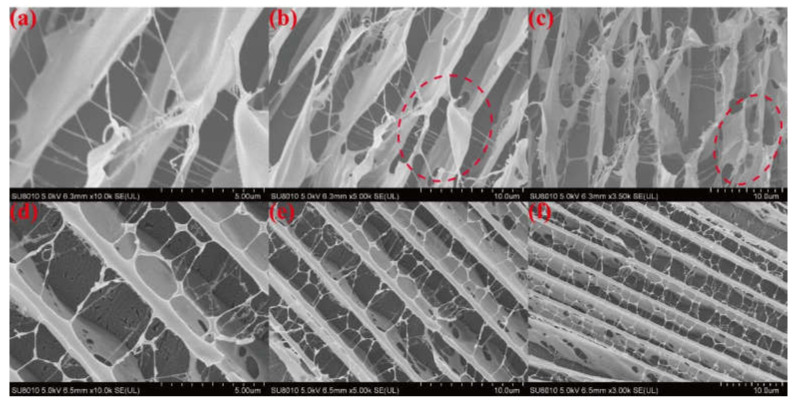
Cryo-SEM images of polymer solutions with and without nano-SiO_2_: polymer solution containing 1 wt% nano-SiO_2_: (**a**) × 10.0 k, (**b**) × 5.00 k, and (**c**) × 3.50 k; polymer solution without nano-SiO_2_: (**d**) × 10.0 k, (**e**) × 5.00 k, and (**f**) × 3.00 k.

The distribution of polymer solutions with low concentrations may be different from that of polymer solutions with high concentrations, so we observed the microstructure of polymer solutions using cryo-SEM to verify the results of the DLS tests. Figure 7 illustrates the microstructures of the 0.6% HPAM solution and the mixture of 1 wt% nano-SiO_2_ and 0.6% HPAM. Without SiO_2_ nanoparticles in the solution (Figure 7a–c), the polymer was dispersed in an aqueous solution in the form of scattered sheets, and there was obvious entanglement between the polymer molecular chains (as shown in the red circles). After the addition of nano-SiO_2_ to the solution, the dispersion state of the polymer in water became tidier, the polymer molecules showed a uniform layer-like distribution, and even the molecular chain distances tended to be the same (Figure 7d–f). The entanglement between polymer molecules was also significantly reduced. Therefore, the addition of nano-SiO_2_ to a highly concentrated polymer solution could also contribute to the homogeneous dispersion of the solution.

**Figure 8:**
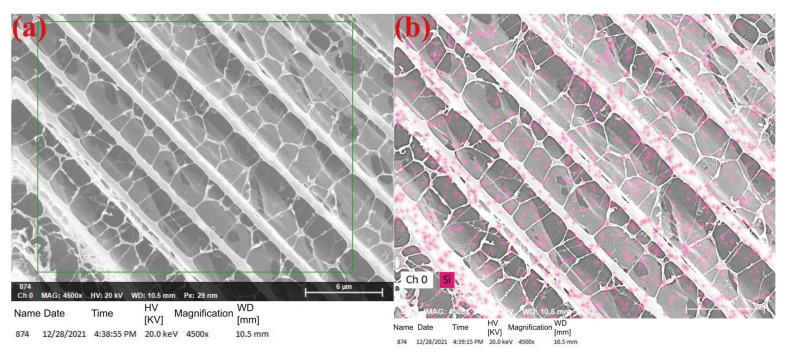
Distribution of nano-SiO_2_ in polymer solution: (**a**) scanning region; (**b**) silicon element distribution.

Figure 8 presents the energy spectrum of the distribution of Si elements in the mixture of nano-SiO_2_ and HPAM. The distribution of Si elements was more concentrated near the polymer molecular chains, indicating that nano-SiO_2_ may be adsorbed on the polymer molecular chains through hydrogen bonding [23,28], thus effectively inhibiting the interactions between polymer molecular chains and reducing the entanglement between polymer molecules to some extent.

**Figure 9:**
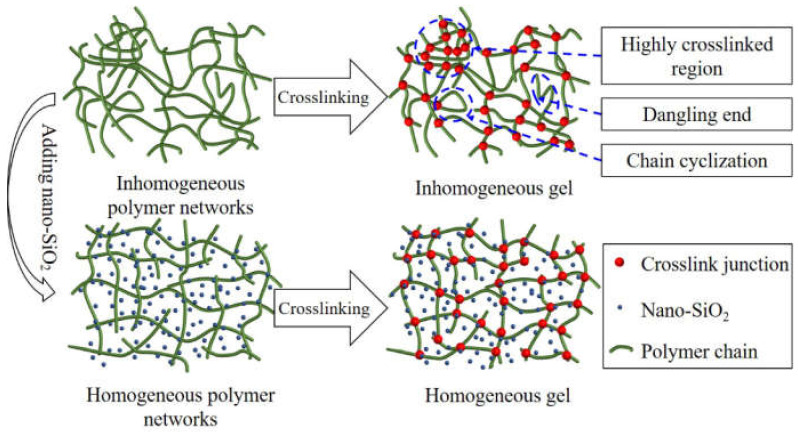
Mechanism of gel strengthening by nano-SiO_2_.

Usually, the formed gels show a non-homogeneous network state with chain cyclization, dangling ends, and highly crosslinked regions within the gel network (Figure 9). The structures of chain cyclization and dangling ends contribute little to the strength of the gel, whereas the highly crosslinked regions make the gel locally stiff and also cause a relative decrease in crosslink density in other regions of the gel. In addition, the molecular chains between the two crosslinked junctions in the highly crosslinked region were shorter, and the grid space was tighter, so its elastic deformation capacity and water-holding capacity would be weakened [31,34,35]. Fortunately, the addition of nano-SiO_2_ improved the dispersion of HPAM in the gelling solution, resulting in a more homogeneous gel network structure and a reduction in the number of structures that are unfavorable to gel strength, as mentioned above, resulting in higher gel strength. Figure 9 shows a schematic diagram of the mechanism of nanoparticle enhancement of the gel strength by modifying the dispersion of the polymer.

#### 2.2.3. Effect of Nano-SiO_2_ on the Thermal Stability of Polymers

**Figure 10:**
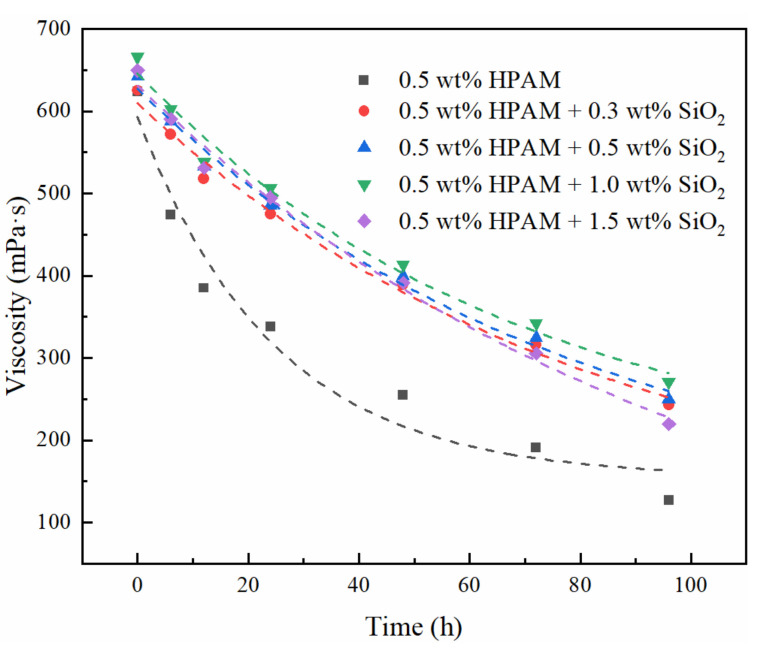
Viscosity of polymer solutions aged at 90 °C for different times.

In this study, by adding nano-SiO_2_ to the gelant, the thermal stability time of the formed gel was dramatically increased from 5 days to more than 180 days at 110 °C. This mechanism may be related to the improved thermal stability of the polymer by nanoparticles. The effect of nano-SiO_2_ on the thermal stability of the gels was characterized by measuring the viscosity of solutions containing 0.5% HPAM and different concentrations of nano-SiO_2_ aged at high temperatures for different times. Because the temperature resistance of the uncrosslinked HPAM is limited, even nano-SiO_2_ may fail to inhibit its rapid degradation at 110 °C. Therefore, we fixed the aging temperature at 90 °C; the results are shown in Figure 10.

The addition of nano-SiO_2_ significantly increased the viscosity of the polymer solution at room temperature, and the solution viscosity was highest at a nano-SiO_2_ concentration of 1%. Moreover, the addition of nano-SiO_2_ significantly retarded the decreasing trend of the viscosity of the polymer during aging at high temperatures, and the viscosity retention (defined as the ratio of the viscosity of the polymer solution after aging to that before aging) increased significantly after a period of aging. The viscosity retention of polymer solutions containing 0, 0.3 wt%, 0.5 wt%, 1.0 wt%, and 1.5 wt% nano-SiO_2_ after 96 h aging at 90 °C were 20.3%, 38.8%, 38.9%, 40.7%, and 33.8%, respectively. The increased viscosity and thermal stability of the polymer solution suggest that nano-SiO_2_ does interact with the polymer, increasing the strength of the polymer network and inhibiting its degradation. Combined with the concentrated distribution of nano-SiO_2_ near the polymer chains shown in Figure 8, we suggest that nano-SiO_2_ adsorbs on polymer molecules through hydrogen bonding to increase the thermal stability and strength of the gel, which has been widely recognized by researchers [20,21,22,24].

### 2.3. Early Dehydration Mechanism of Gel Aging

**Figure 11:**
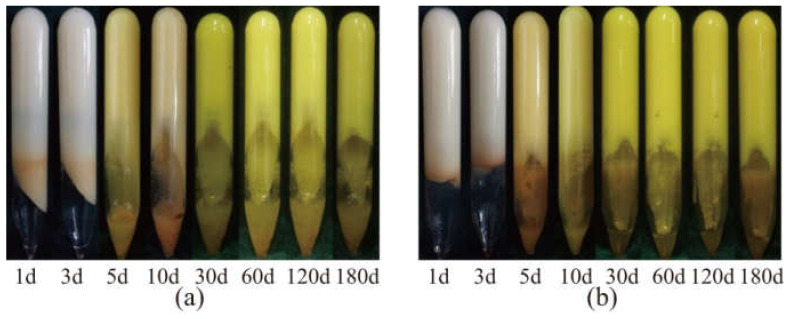
Aging status of gels with different compositions at 110 °C for different times: (**a**) gel prepared with 0.4 wt% HPAM, 0.4 wt% WSPR, and 1.0 wt% nano-SiO_2_; (**b**) Gel prepared with 1.0 wt% HPAM, 1.0 wt% WSPR, and 1.0 wt% nano-SiO_2_.

Figure 11 presents the states of two gels after aging at 110 °C for different times.

**Figure 12:**
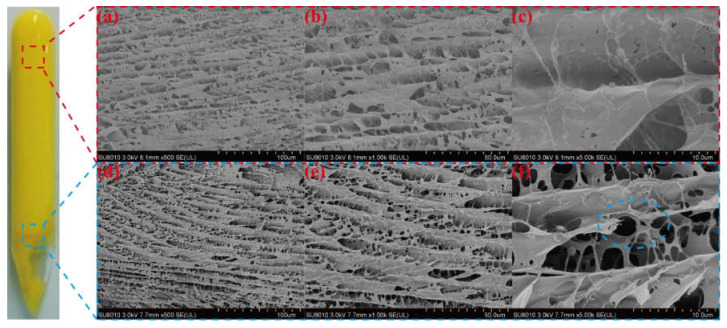
Cryo-SEM images of different parts of early dehydrated gels: microstructure of the gel at the bottom of the ampoule: (**a**) × 500, (**b**) × 1.00 k, and (**c**) × 5.00 k; microstructure of the gel on top of the ampoule: (**d**) × 500, (**e**) × 1.00 k, and (**f**) × 5.00 k.

The gel dehydration state can be divided into three stages: the slow dehydration period (1–3 days), rapid dehydration period (3–5 days), and stabilization period (after 5 days). For example, the dehydration ratio of the gel composed of 1.0 wt% HPAM, 1.0 wt% WSPR, and 1.0 wt% nano-SiO_2_ was 9.8% when aged at 110 °C up to 5 days, whereas the dehydration ratio was almost stable with continued aging, and the dehydration ratio was only 10.9% when aged up to 180 days. Although the phenomenon of dehydration during the first 5 days of gel aging (the so-called early dehydration of gel aging) was suppressed by increasing the concentration of HPAM, WSPR, and nano-SiO_2_, although the effect was very limited. We believe that this phenomenon may be related to the oxidative degradation of the polymer. Because the gel was not deoxygenated prior to aging, it contained a certain amount of dissolved oxygen, and oxygen was also present in the air at the top of the ampoule. This oxygen could cause oxidative degradation of the polymer, leading to disruption of the gel structure and consequent dehydration of the gel. The dehydration ratio no longer changed after 10 days, so it can be assumed that the oxygen in the ampoule was consumed. Cryo-SEM images of different parts of the gel in the presence of early dehydration were observed (Figure 12) to verify this hypothesis. We found that the gel network at the bottom of the ampoule was dense, with a complete structure and good continuity between molecular chains (Figure 12a–c), whereas the gel network at the top of the ampoule in contact with air had a looser structure, with porous networks and some broken polymer links (Figure 12d–f). These features can be thought of as more oxidative degradation of the polymer chains of gels exposed to air, resulting in the destruction of the gel structure and a decrease in water-holding capacity, leading to early dehydration.

**Figure 13:**
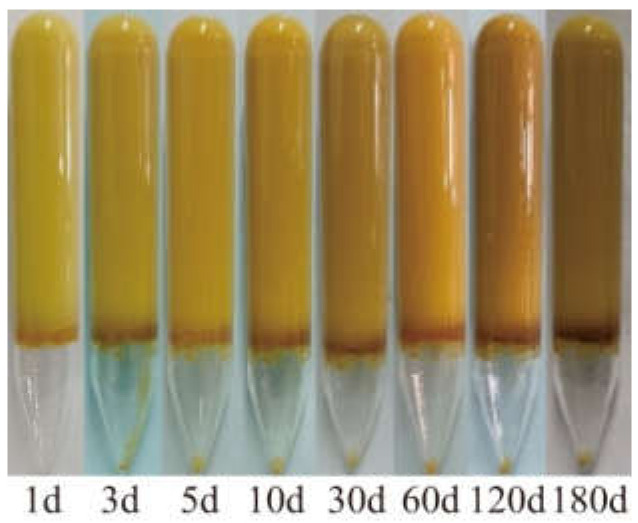
The state of gels formed by 1 wt% HPAM, 1 wt% WSPR, 1 wt% nano-SiO_2_, and 0.025 wt% HQ aged at 110 °C for different times.

The above results indicate that nanoparticles cannot completely inhibit the oxidative degradation of the polymer. Because it is difficult to provide measures for deoxidation in the practical application of gels, a method to inhibit gel degradation is needed. As the amide groups of HPAM cannot all participate in the crosslinking reaction, the remaining amide groups are very susceptible to oxidative degradation. Therefore, we added a small amount of HQ to the gel as a free-radical terminator that can react with the amide groups that are not involved in crosslinking. Furthermore, HQ is easily oxidized, which also consumes some of the oxygen in the gelling agent. As shown in Figure 13, the addition of a small amount of HQ significantly improved the dehydration of the gel and did not affect its stability.

## 3. Conclusions

1. High-strength gel was prepared with HPAM as the main agent, WSPR as the crosslinker, and nano-SiO_2_ as the stabilizer and was stable for more than 180 days at 110 °C in brine with a salinity of 12.124 g/L, with an adjustable gelation time of between 3 and 23 h, although there was a certain degree of early dehydration.
2. Nano-SiO_2_ can effectively improve the dispersion of polymers in aqueous solutions. By inhibiting the entanglement and aggregation of the polymer, a more homogeneous lattice structure is formed, which, in turn, results in a stronger gel.
3. The significant interaction between nano-SiO_2_ and polymers can enhance the gel structure and inhibit the degradation of polymers to a certain extent, which has the effect of improving the gel strength and thermal stability.
4. The early dehydration of WSPR gels reinforced with nano-SiO_2_ is caused by the oxidative degradation of the polymer, which can be improved by adding a small amount of HQ to the system.

## 4. Materials and Methods

### 4.1. Materials

The main agent of the gel was partially hydrolyzed polyacrylamide (HPAM) with a 25% hydrolysis degree and an average molecular weight of 12 × 10^6^ g/mol supplied by Hebei Liancheng Environmental Protection Technology Co., Shijiazhuang, China. The crosslinker was a water-soluble phenolic resin (WSPR) solution with an effective content of 30% provided by Dongying Tongkun Technology Co., Dongying, China. The nano-SiO_2_ solution with an effective solid content of 30% and a median particle size of 15 nm for nanoparticles was purchased from Dongying Liuhe Co., Dongying, China. Analytically pure hydroquinone provided by Sinopharm Chemical Reagent Co., Shanghai, China, was also used in the experiment. In addition, analytically pure drugs, such as sodium chloride, potassium chloride, anhydrous calcium chloride, magnesium chloride hexahydrate, sodium bicarbonate, and sodium sulfate from Sinopharm Chemical Reagent Co., Shanghai, China, were used in this study to prepare synthetic saline for the preparation of gels.

To simulate the salinity conditions of formation water in the reservoir, a synthetic brine with a salinity of 12.124 g/L was synthesized using inorganic salts to formulate a gelant. The composition of the synthetic brine is shown in Table 2.

### 4.2. Methods

#### 4.2.1. Preparation of Gels

The gels were prepared at room temperature without removing oxygen. First, synthetic brine, nano-SiO_2_, and WSPR were weighed according to the pre-designed gel formulation and mixed in a beaker. The mixture was stirred well using a glass rod and placed under a stirrer. Then, the polymer was slowly added to the solution while stirring at 300 r/min. Subsequently, the solution was stirred at 120 r/min for 4 h to produce a homogeneous gelant. Finally, 20 g of gelant was injected into an ampoule that was be sealed and placed in an oven at 110 °C. Multiple identical samples of each gel were prepared for systematic evaluation and comparison of gel performance.

#### 4.2.2. Evaluation of Gels

The performance of the gels was evaluated mainly through the measurement of gelation time, gel strength, and thermal stability. (1) The gelation time was determined by the gel strength codes as defined by Sydansk [36]. In this paper, the time elapsed for the gel to reach the F level was defined as the gelation time. (2) The gel strength was characterized by the storage modulus in the linear viscoelastic region of the gel measured by a rheometer (MCR 92, Anton Paar) equipped with a parallel-plate geometry system at 25 °C. In the test, the strain amplitude ranged from 1% to 1000%, whereas the oscillation frequency was fixed at 1Hz, and the parallel plate gap was fixed at 1mm. (3) The thermal stability of the gel was characterized by determining the dehydration ratio of the gel aging at high temperatures for different times. The dehydration ratio was defined as the ratio of the mass of water removed from the gel to the mass of the original gelant.

#### 4.2.3. Dynamic Light Scattering (DLS) Analysis

The hydrodynamic radii (R_h_) of the polymers in nano-SiO_2_ solutions were determined by a particle size analyzer (90Plus PALS, Brookhaven) at 25 °C. The nano-SiO_2_ solutions were diluted to different concentrations using distilled water. HPAM was subsequently added to the different concentrations of nano-SiO_2_ solutions while stirring, and the solutions were stirred to homogeneity using a stirrer at 120 r/min. The prepared solution was sealed and left to stand at 25 °C for 24 h and then tested for R_h_. The R_h_ distribution of each sample was measured five times, and the log-normal distribution curve of the R_h_ was calculated using particle size analysis software. It should be noted that polymers have very long molecular chains that may become entangled in aqueous solutions and produce polymer clusters with hydrodynamic radii beyond the measurement range of the instrument. Therefore, the polymer concentration in the hydrodynamic radius test was fixed at a low value of 0.05 wt%.

#### 4.2.4. Cryo-Scanning Electron Microscopy (Cryo-SEM) Analysis

The microscopic morphology of polymer solutions and gels was obtained using a cryo-SEM. The sample morphology was maintained by rapid freezing using liquid nitrogen at −160 °C, followed by vacuum drying. The samples were then sprayed with nanogold and placed on the electron microscope stage for observation and imaging.

#### 4.2.5. Rheology Tests

The viscosity of the polymer solution was determined by a rheometer (MCR 92, Anton Paar) equipped with a coaxial cylinder geometry (the inner diameter of the sample cup was 42 mm, and the diameter of the rotor was 39 mm) at 25 °C. Polymer solutions with different nano-SiO_2_ concentrations were prepared using the same method described in Section 4.2.3. The viscosity was measured after the samples were sealed without de-oxygenation and aged at 90 °C for different times. The tests were carried out in a controlled shear rate model with the shear rate fixed at 7.34 s^−1^.

## Figures and Tables

**Table 1:** Recent references on enhanced gels.

Authors, Year, and References	Polymer	Crosslinker	Nanoparticles	Proposed Nanoparticle-Enhanced Gel Mechanism
Dai et al. (2016) [19]	Polyacrylamide (PAM)	Phenolic resin	Silica	Nanoparticles strengthen the gel mesh and retard polymer degradation.
Yang et al. (2021) [20]	HPAM	Organic zirconium	Silica	Nanoparticles can increase the network density and adsorb on the polymer to enhance the chain strength.
Liu et al. (2017) [21]	PAM	HQ, HMTA	Silica	Nanoparticles can increase the mesh density and the polymer chain strength. Hydrogen bonding and electrostatic attraction of nanoparticles with water increase the hydrophilicity and thermal stability of the gel.
Chen et al. (2018) [22]	PAM	Polyethylenimine (PEI)	Silica	Nanoparticles increase the density and strength of the mesh by crosslinking with the polymer through hydrogen bonding.
Shamlooh et al. (2019) [23]	PAM	PEI	Silica	Hydrogen bonding between the nanosilica and the polymer is the key to the formation of a stable network.
Giraldo et al. (2017) [24]	HPAM	/	Silica	Nanoparticles can adsorb on polymers to inhibit their degradation.
Zhu et al. (2014) [25]	Hydrophobically associating polyacrylamide	/	Silica	Nanoparticles are physically crosslinked with the polymer through hydrogen bonding to enhance the strength and thermal stability of the mesh.
Zareie et al. (2019) [26]	HPAM	Chromium acetate	Silica	Nanoparticles can be added to the gel mesh as fillers to increase the gel strength.
Shamlooh et al. (2020) [27]	Polyacrylamide tert-butyl acrylate	PEI	Silica	Nanoparticles generate physically crosslinked enhanced gels with the polymer through hydrogen bonding.

**Table 2:** Composition of simulated formation water.

	Ions						Total Dissolved Solids
	Na^+^, K^+^	Mg^2+^	Ca^2+^	Cl^−^	SO_4_^2−^	HCO_3_^−^	
Content (g/L)	4.121	0.038	0.047	4.66	0.012	3.243	12.124

## Data Availability

The data generated and analyzed during this study are available from the corresponding author upon reasonable request.
